# Inhibition of phosphorus removal performance in activated sludge by Fe(III) exposure: transitions in dominant metabolic pathways

**DOI:** 10.3389/fmicb.2024.1424938

**Published:** 2024-06-12

**Authors:** Yiyihui Hong, Hong Cheng, Xiaoliu Huangfu, Lin Li, Qiang He

**Affiliations:** Key Laboratory of the Three Gorges Reservoir Region’s Eco-Environment, Ministry of Education, College of Environment and Ecology, Chongqing University, Chongqing, China

**Keywords:** wastewater treatment, simultaneous chemical phosphorus removal, iron salts, biological toxicity, polyphosphate-accumulating metabolism

## Abstract

**Introduction:**

Simultaneous chemical phosphorus removal process using iron salts (Fe(III)) has been widely utilized in wastewater treatment to meet increasingly stringent discharge standards. However, the inhibitory effect of Fe(III) on the biological phosphorus removal system remains a topic of debate, with its precise mechanism yet to be fully understood.

**Methods:**

Batch and long-term exposure experiments were conducted in six sequencing batch reactors (SBRs) operating for 155 days. Synthetic wastewater containing various Fe/P ratios (i.e., Fe/P = 1, 1.2, 1.5, 1.8, and 2) was slowly poured into the SBRs during the experimental period to assess the effects of acute and chronic Fe(III) exposure on polyphosphate-accumulating organism (PAO) growth and phosphorus metabolism.

**Results:**

Experimental results revealed that prolonged Fe(III) exposure induced a transition in the dominant phosphorus removal mechanism within activated sludge, resulting in a diminished availability of phosphorus for bio-metabolism. In Fe(III)-treated groups, intracellular phosphorus storage ranged from 3.11 to 7.67 mg/g VSS, representing only 26.01 to 64.13% of the control. Although the abundance of widely reported PAOs (*Candidatus Accumulibacter*) was 30.15% in the experimental group, phosphorus release and uptake were strongly inhibited by high dosage of Fe(III). Furthermore, the abundance of functional genes associated with key enzymes in the glycogen metabolism pathway increased while those related to the polyphosphate metabolism pathway decreased under chronic Fe(III) stress.

**Discussion:**

These findings collectively suggest that the energy generated from polyhydroxyalkanoates oxidation in PAOs primarily facilitated glycogen metabolism rather than promoting phosphorus uptake. Consequently, the dominant metabolic pathway of communities shifted from polyphosphate-accumulating metabolism to glycogen-accumulating metabolism as the major contributor to the decreased biological phosphorus removal performance.

## 1 Introduction

The sources and fate of phosphorus from municipal wastewater treatment plants (WWTPs) play crucial roles in phosphorus biogeochemical cycling, contributing to the improvement of environmental function quality. Currently, the simplest and cost-effective method for phosphorus removal in WWTPs is enhanced biological phosphorus removal (EBPR). However, the susceptibility of EBPR to external environmental factors such as temperature, pH, and hydraulic retention time (HRT) often leads to unstable phosphorus removal, with effluent phosphorus concentrations ranging from 0.5 to 1 mg-P/L (Mino et al., 1998; Hussain et al., 2011; Di Capua et al., 2022). Consequently, simultaneous chemical phosphorus removal (SCPR) is emerging as an attractive and increasingly popular alternative (Zhang et al., 2015). According to a previous report, WWTPs in China treat approximately 0.31 million tons of phosphorus annually, of which SCPR accounted for more than 43.5% (China Ministry of Ecology and Environment, 2023). Particularly, iron salts (Fe(III)) stand out as the most prominent chemical agents for phosphorus removal due to their distinct advantages, such as reduced hydrogen sulfide emissions and enhanced sludge settling and dewatering properties.

Previous toxicological studies have indicated that Fe(III) dosing has an inhibitory effect on biological phosphorus removal of activated sludge (AS), however, its inhibitory mechanism has not been well revealed (Liu et al., 2011; Zhang et al., 2018). Firstly, AS is a highly complex system with significant biological diversity. Though *Candidatus Accumulibacter* is generally regarded as the most dominant genus of polyphosphate accumulating organisms (PAOs), recent research has demonstrated that *Dechloromonas*, *Thiothrix*, *Comamonadaceae*, and *Tetrasphaera* also exhibit considerable phosphorus metabolic activity (Zhao et al., 2017; Yan et al., 2022). In particular, *Tetrasphaera* is frequently detected in wastewater, even at higher levels than *Candidatus Accumulibacter* (Stokholm-Bjerregaard et al., 2017). Consequently, understanding the potential impact of Fe ions on AS in WWTPs solely based on its toxicity to *Candidatus Accumulibacter* is challenging. Secondly, as an inevitable pathway of mass transfer between cells and the external environment, the effect of extracellular polymeric substances (EPS) on phosphorus uptake and release cannot be overlooked. Fe ions inhibit the growth of PAOs by interfering intracellular polyhydroxyalkanoates (PHAs) synthesis and degradation, while EPS can partially protect the bacterial cell from damage (Li et al., 2015; Zhang P. et al., 2019; Peng et al., 2024). However, there are still controversies regarding the specific role of EPS in the presence of metal ions (Shi et al., 2017). Thirdly, glycogen accumulating organisms (GAOs) share similar metabolic characteristics with PAOs but lack phosphorus removal capacity. In general, excessive Fe(III) reacts chemically with phosphate (e.g., chemiprecipitation or chemisorption), resulting in a high C/P ratio within the system, which can promote GAOs proliferation and reduce phosphorus removal efficiency (Welles et al., 2015). But existing studies have indicated that PAOs persist in AS systems even in the presence of high metal salt dosage (Wang et al., 2020). As a result, it cannot be ruled out that PAOs indeed change their metabolic strategy in order to survive the Fe(III) stress.

The uncertainty regarding these potential mechanisms led to overdosing of chemical reagent and low chemical phosphorus removal efficiency in practice (Liu et al., 2011). Hence, it is imperative to elucidate more aspects of the influencing mechanism of the Fe(III) on biological phosphorus removal. In this study, six parallel sequencing batch reactors (SBRs) with EBPR ability were operated and ferric chloride was selected as the representative chemical phosphorus removal reagent. The main objectives are to (1) investigate the biological phosphorus removal behavior under varying Fe(III) dosages, (2) evaluate the variations of the phosphorus removal mechanism in AS induced by Fe(III) dosing, and (3) reveal the inhibitory mechanism on biological phosphorus removal resulting from prolonged Fe(III) stress. This study attempts to offer new insights into the inhibitory mechanism of Fe(III) on biological phosphorus removal and provide new perspectives for SCPR process optimization in practice.

## 2 Materials and methods

### 2.1 Experimental reactors configuration and operation condition

Laboratory-scale SBRs were made of cylindrical Plexiglas with a working volume of 2.5 L. The SBR system was designed with an average HRT of 12 h and a solids retention time (SRT) of 10 days. Schematic diagram of the SBR and operational details are available in the Supplementary Figure S1 and Supplementary Text S1. Prior to the experiments, the anionic surfactant sodium dodecyl sulfate was added and mixed into the seed sludge suspension obtained from a full-scale wastewater treatment plant (WWTP) in Chongqing, China, and a final concentration was 60 mg/g SS (Shi et al., 2021). The mixture was stirred at 150 r/min for 10 min to remove chemical flocculants. The mixture was washed several times until Fe was undetectable in the eluate. Synthetic wastewater was then injected for sludge acclimation to elicit microbial activity. It has been reported that the Fe/P molar ratio in the SCPR system can range from 1.25 to 1.9 (Clark et al., 2000; Caravelli et al., 2010). Correspondingly, synthetic wastewater containing various Fe/P ratios (i.e., Fe/P = 1, 1.2, 1.5, 1.8 and 2) was slowly poured into the SBRs during our experiment, designated as R2, R3, R4, R5, and R6, respectively. Control experiments were performed by pumping equal volumes of deionized water into influent (denoted as R1).

The six bioreactors were continuously operated for 155 days, divided into three phases. Phase 0, spanning from day 0 to day15, served as the culture acclimatization period to obtain stable contaminant removal efficiency and microbial community differentiation. Phase I, extending from day 16 to day 55, was the initial adjustment period aimed at observing the acute response of AS to Fe(III) addition. Phase II, spanning from day 56 to day 165, represented the long-term stabilization period during which the effects of chronic Fe(III) exposure on PAO and phosphorus metabolism were evaluated. All reactors were operated at room temperature (25 ± 2°C) with four 6 h cycles per day. Each cycle included feeding (5 min), anaerobic mixing (115 min), aerobic aeration (180 min), sludge withdrawing (5 min), settling (25 min), supernatant withdrawing (20 min), and idling (10 min). These steps were sequentially controlled by a programmable logic controller. Detailed operating procedures and influent composition are provided in Supplementary Text S2 and Supplementary Tables S1, S2.

### 2.2 Separation of EPS and bacterial cells

To assess the possible effects of Fe(III) on intracellular and extracellular bio-phosphorus, crude EPS extraction and collection of microbial cells were conducted from AS samples using a cation exchange resin method modified by Long et al. (2017). After filtering out the resin particles, the mixture was centrifuged for solid–liquid separation at 5,000 r/min for 10 min, and the supernatant and precipitate were collected. The supernatant was regarded as the total EPS solution, and the centrifuged precipitate was considered as the bacterial cell extract (Tao et al., 2020). The supernatant collected after filtration with a 0.22 μm filter was the EPS solution to be tested. The precipitate was oven-dried to a constant dry weight at 80°C, and then calcined at 500°C for 4 h to remove the organic compounds. Subsequently, the obtained grey precipitate was acid-digested with H_2_SO_4_/HClO_4_ mixture (1/4, v/v), and completely washed with deionized water. Fe and phosphorus concentrations in samples were determined after filtration with a 0.22 μm filter.

### 2.3 Intracellular PHAs analysis

To determine the conversion rates of metabolic intermediates under different Fe(III) dosages, total PHA (including poly-β-hydroxybutyrate (PHB), poly-β-hydroxyvalerate (PHV), and poly-β-2-hydroxymethylvalerate (PH2MV)) in the samples were quantified using gas chromatography (GC) with benzoic acid as the internal standard, following the method described by Oehmen et al. (2005). Thirty millilitres of fresh sludge mixture was collected and mixed with formaldehyde to inhibit the bioactivity of this AS. After high-speed centrifugation (8,000 g for 5 min), the precipitate was freeze-dried. A 30 mg sample of freeze-dried sludge was then mixed with 2 mL of chloroform and 2 mL of acidified methanol (PHB and PHV: 4%, PH2MV: 10%) in a 10 mL digestion tube. The mixture was digested at 100°C for 20 h. After cooling to room temperature, 1 mL of distilled water was added into the mixture, and 1.5 mL of metabolites were sampled from the chloroform phase.

The products were analyzed using GC on an Agilent 6890 instrument with a RTX-5 capillary column (1 μm × 0.32 mm × 50 m), employing high purity nitrogen as the carrier gas and a flame ionization detector. The injector temperature was maintained at 250°C. The oven temperature was initially set at 120°C for 2 min, then ramped up at a rate of 8°C/min until reaching 200°C for 2 min, followed by a further increase at 15°C/min up to 270°C for 2 min.

### 2.4 Key enzymes activities assay

The activity of key intracellular enzymes directly affects phosphorus removal and enrichment by microbes. To assess the effects of Fe(III) on phosphorus and polyphosphate (Poly-P) metabolism in AS, the polyphosphate kinase (PPK) and exopolyphosphatase (PPX) activities were determined. For sample preparation, 30 mL of sludge mixture collected at the end of anaerobic mixing was utilized. Homogenized samples were subsequently centrifuged at 6,000 g for 10 min at 4°C. The resulting precipitates were washed three times with 1.5 M NaCl buffer (containing 0.01 M EDTA and 1 mM NaF, pH 7.4) and were then resuspended in 30 mL of loading buffer (Long et al., 2017). Subsequently, the samples were sonicated at 4°C for 5 min (3 s sonication and 5 s break), and cellular debris was removed through centrifugation (12,000 g, 4°C, and 10 min) to obtain the crude extracts.

For the PPK assay, 150 μL of crude extracts were mixed with a reaction solution (850 μL) containing 100 mM Tris-HCl (pH 7.4), 8 mM MgCl_2_, 200 mM D-glucose, 0.5 mM NADP, 150 μg of Poly-P, 1 unit of hexokinase, and 1 unit of glucose-6-phosphate dehydrogenase (Zheng et al., 2011). The reaction was initiated by adding 1 mM ADP to each sample, followed by incubation in a water bath at 37°C for 45 min, the NADPH production was determined by a spectrophotometer at 340 nm.

In parallel, for the PPX assay, 0.5 mL of crude extracts were combined with 2 mL of PPX reaction solution containing 0.5 M Tris-HCl buffer (pH 7.4), 5 mM MgCl_2_, and 2.5 mM p-nitrophenyl phosphate. The reaction mixtures were incubated in a water bath at 30°C for 45 min, and the reaction was halted by adding 2 mL of 0.5 M KOH. The production of p-nitrophenol was determined by a spectrophotometer at 405 nm.

### 2.5 Metagenomic analysis

To further elucidate the mechanism of the transition in phosphorus removal metabolic pathways under Fe(III) stress, the composition and metabolic potential of functional bacterial communities were predicted by analyzing 16S rRNA and synthase gene sequences in metagenomic reads. Briefly, total genomic DNA was extracted from freeze-dried sludge using a PowerSoil DNA extraction kit (Qiagen) following the manufacturer’s instructions. After purification, Paired-end metagenomics libraries were prepared using an Illumina TruSeq DNA Library Preparation Kitand and sequenced on a HiSeq 4000 platform for 350 bp sequencing by Majorbio Bio-Pharm Technology Corp. (Shanghai, China). Each sample yielded at least 5.8 GB of total reads, and all raw sequencing reads have been deposited in the NCBI Sequence Read Archive (PRJNA1104697).

Detailed steps of the metagenomic analysis are provided in Supplementary Text S3. Following quality control and sequences assembly, high quality sequencing reads (95% identity and 90% coverage) were aligned to the reference AS genome sequence (Hong et al., 2022). The abundance of each gene was calculated as mapped reads per kilobase per million (RPKM) total reads in the library. Subsequently, representative sequences of the non-redundant gene catalog were aligned to NCBI NR and KEGG database using BLASTP (with an e-value cutoff of ≤10^−5^) to obtain detailed information for taxonomic annotations and potential functions.

### 2.6 Other analytical methods

Wastewater phosphate, total suspended solids, and volatile suspended solids were quantified following the standard methods (American Public Health Association, 2017). The extraction of phosphorus and Fe fractions from sludge samples was conducted using a modified method proposed by Zhang B. et al. (2019). The Fe element level in each sample was determined using inductively coupled plasma optical emission spectroscopy (ICP-OES, Optima 8000, PerkinElmer, United States). To minimize cellular disruption, EPS was extracted using a cation exchange resin method as described in previous studies, and the protein and polysaccharide content of the EPS was analyzed (Zhang et al., 2013). Protein content was assessed using a modified Lowry method, while total polysaccharide content was determined using the sulfuric acid-anthrone colorimetric method. Cell membrane integrity in EPS extracts was evaluated using a lactate dehydrogenase (LDH) detection kit, as directed by the manufacturer.

Continuous variables were compared using the two-tailed unpaired Student’s t-test, and multiple group variables were analyzed using one-way analysis of variance (ANOVA). Results were presented as the mean (± standard deviation (SD)), with differences considered significant at *p*-value ≤0.05.

## 3 Results

### 3.1 Phosphorus removal in Fe(III)-treated AS

**Figure 1:**
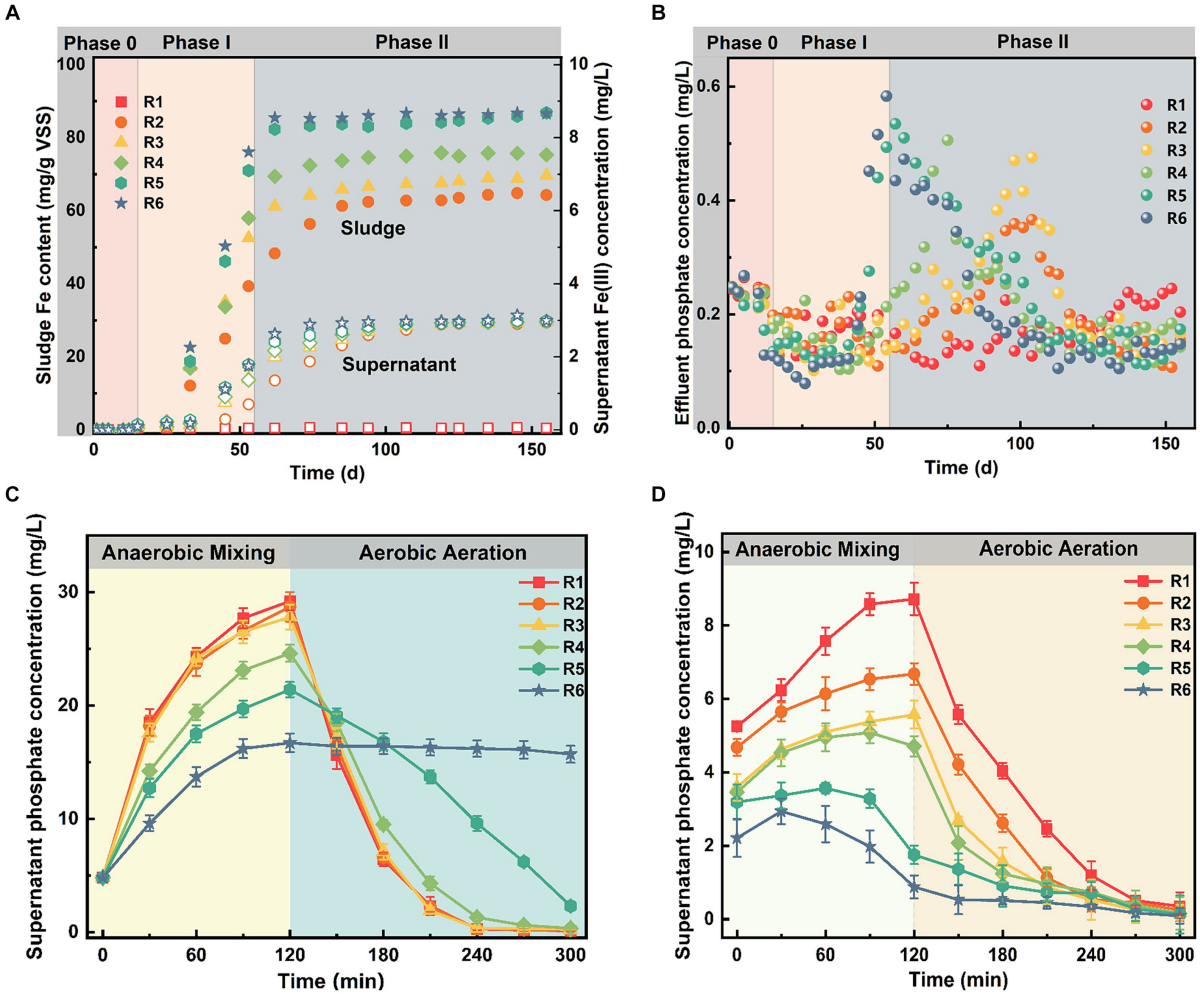
The long-term variations in **(A)** iron levels in the mixture and **(B)** phosphate concentrations in the effluent, and the dynamic distribution of supernatant phosphate concentrations at the end of **(C)** Phase I and **(D)** Phase II. Error bars represent the standard deviation from three independent replicates.

The variations of soluble Fe content were monitored over long-term exposure. Due to excess Fe(III) dosing, sludge Fe content in experimental groups gradually increased and began to accumulate in the supernatant over time. Specifically, while the sludge Fe content in R1 consistently remained at low levels of 0.22 ± 0.03 mg/g VSS, its levels in R2–R6 began to increase rapidly after continuous Fe(III) dosing for 19 days, as depicted in Figure 1A. In addition, temporal changes in supernatant Fe(III) concentrations in all groups exhibited a similar hysteresis phenomenon. Supernatant Fe(III) concentrations did not change at first until day 31, after which they increased significantly.

Upon Fe(III) addition, phosphate removal efficiency in R2–R6 undergoes three stages: initial stabilization, subsequent deterioration, and eventual re-stabilization (Figure 1B). For instance, the average effluent phosphate concentration in R1 during Phase I was 0.17 ± 0.04 mg/L, with over 98% removal efficiency. Although the average phosphate removal in R2–R6 was slightly higher than that in R1 during the first 30 days following Fe(III) exposure, the difference (within 0.4%) was not statistically significant (*p* > 0.05). However, phosphate removal efficiency in R2–R6 began to deteriorate one by one after day 55, peaking on days 105, 99, 76, 58, and 55, respectively.

To delve into the underlying inhibitory mechanism of Fe(III) on biological phosphorus removal, we meticulously examined sludge mixtures collected at different time points (day 55 and day 155). Figures 1C,D depict the temporal variation of phosphate concentrations during a complete cycle at the end of Phase I and Phase II. Except for R6, all reactors followed a typical trend of biological phosphorus release and uptake at the end of Phase I (Figure 1C). Moreover, no significant difference was noted between treatment and control group in the supernatant phosphate concentrations (*p* > 0.05). The supernatant phosphate concentration at the end of anaerobic stage decreased from 8.72 ± 0.44 mg/L in the control to 0.88 ± 0.31 mg/L in R6 as sludge Fe content increased. In addition, Figure 1D showed that the time taken for supernatant phosphate concentrations to reach maximum levels with increasing sludge Fe content. Particularly in R5 and R6, no phosphorus release occurred during anaerobic mixing. Overall, the inhibition of phosphorus removal efficiency by Fe(III) exposure in SCPR was dose-dependent, and the time it took until performance deterioration shortens with the increase of dosage.

### 3.2 Phosphorus transport and transformation

**Figure 2:**
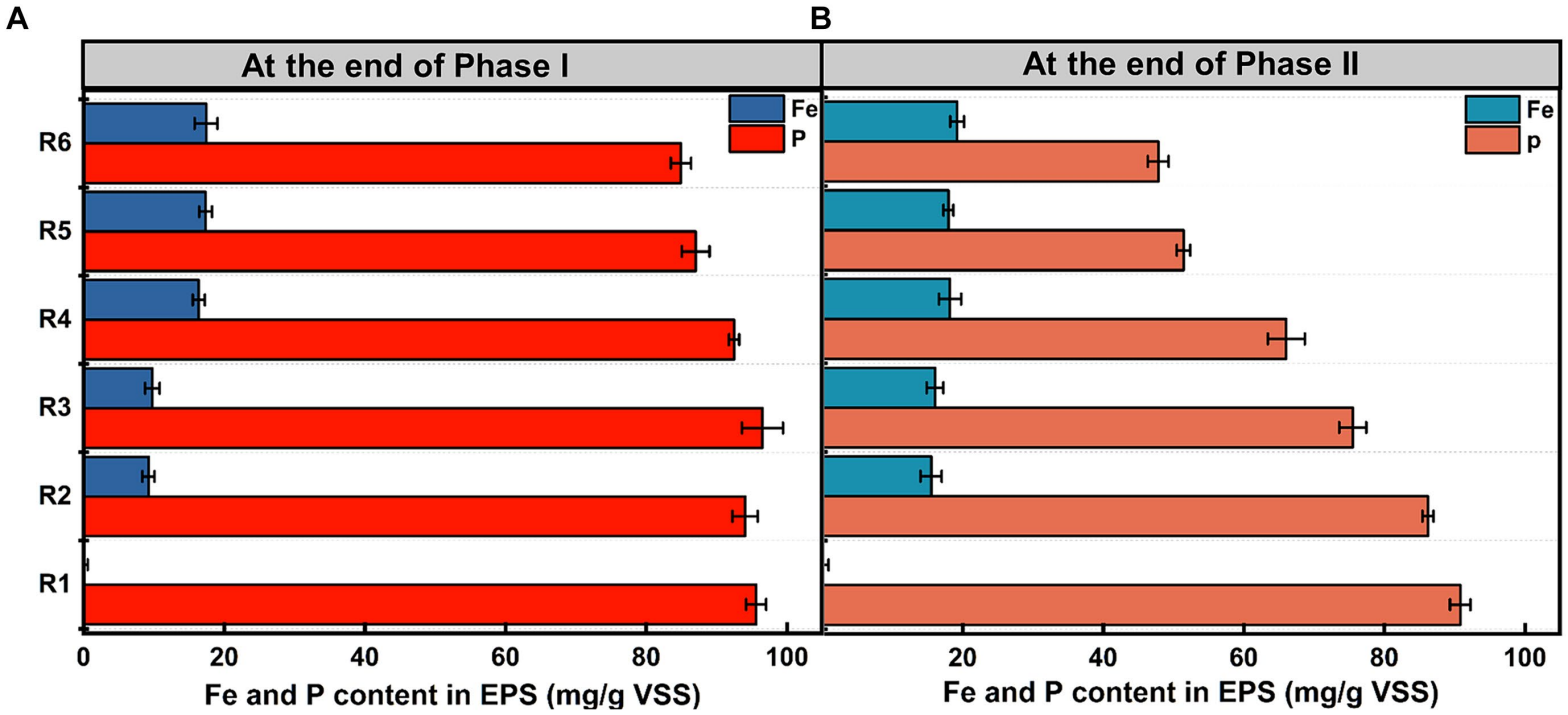
Distribution of phosphorus and Fe levels in EPS at the end of **(A)** Phase I and **(B)** Phase II. Error bars represent the standard deviation from three independent replicates.

Figure 2 illustrates Fe and phosphorus levels in EPS at the end of the anaerobic stage on days 55 and 155. In Phase I (Figure 2A), phosphorus levels in R5 and R6 were only marginally lower than those in the control, with reductions of 8.92 ± 0.21% and 11.19 ± 0.54%, respectively. Differences between other treated groups and the control were not significant (*p* > 0.05). Conversely, in the long-term stability test (Figure 2B), phosphorus levels in tested samples were notably lower than the control. As sludge Fe content increased, phosphorus levels in EPS successively decreased, indicating a strong association with Fe(III) accumulation. However, Fe content in EPS exhibited an opposing trend at the end of Phase I (Figure 2A). An excess of Fe(III) resulted in a large aggregation of Fe species within EPS. At day 155 (Figure 2B), Fe content in treated groups had increased by 55.81 ± 0.66%, 25.44 ± 0.63%, 39.58 ± 1.34%, 10.95 ± 0.79%, and 9.68 ± 1.15%, respectively, compared to the observations in Figure 2A. Fe content of EPS in R5 and R6 remained stable and high level during long-term operation.

**Figure 3:**
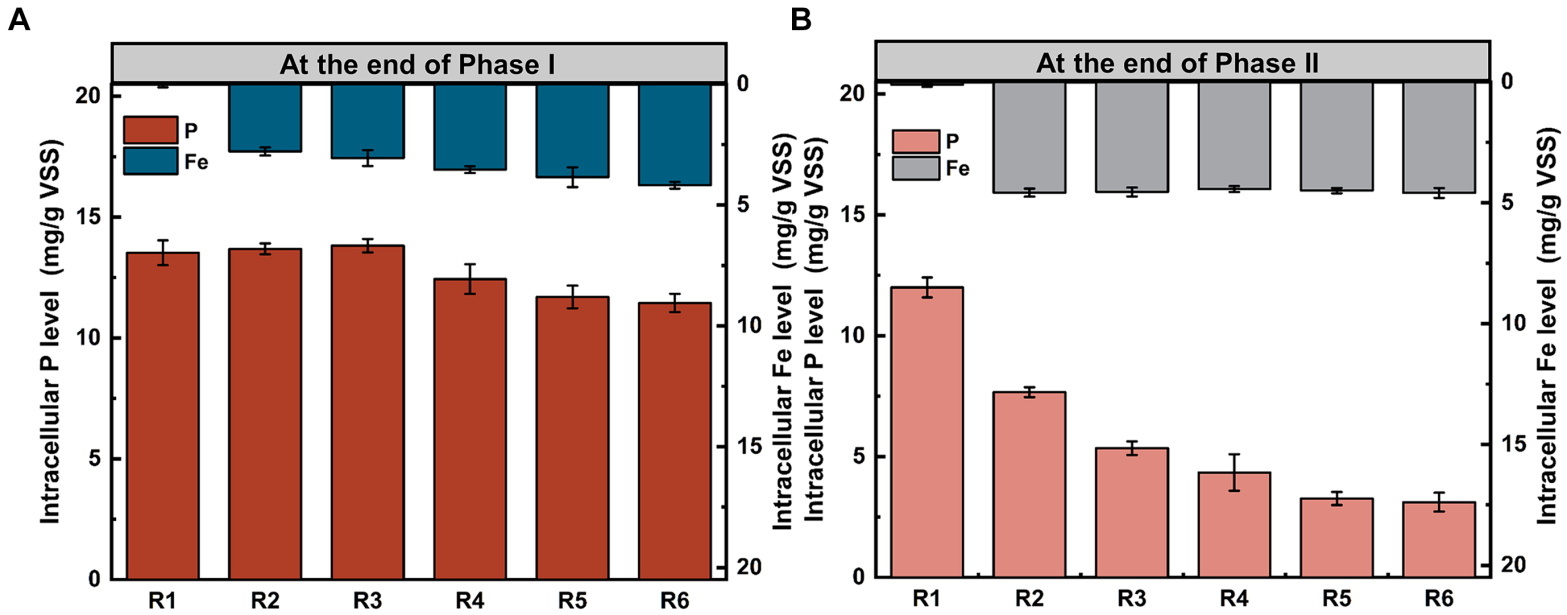
Distribution of phosphorus and Fe levels in PAO cells at the end of **(A)** Phase I and **(B)** Phase II. Error bars represent the standard deviation from three independent replicates.

Additionally, besides being stored in EPS, a portion of phosphorus was transformed into long-chain Poly-P and phosphates within PAO cells. Intracellular phosphorus stocks analyses were conducted for microbial populations at the end of Phase I and Phase II. As Fe content in the mixture increased, intracellular phosphorus stocks initially increased and then decreased (Figures 3A,B). Compared to the control group, intracellular phosphorus stocks in all Fe(III) dosing groups significantly decreased after 155 days of continuous operation (*p* < 0.05). At this point, the intracellular Fe content in the treated groups remained relatively stable with a mean value of 4.53 mg/g VSS. Especially in R6, the intracellular Fe content at the end of Phase II only increased 9.78 ± 0.39% compared to that at the end of Phase I, suggesting that it was probably getting a saturated intracellular concentration. However, there was only a slight increase in cell membrane integrity in the Fe(III)-treated group as compared to that of the control group, but it was not significant (*p* > 0.05) (Supplementary Figure S2).

### 3.3 Intermediates distribution in microbial metabolism

**Figure 4:**
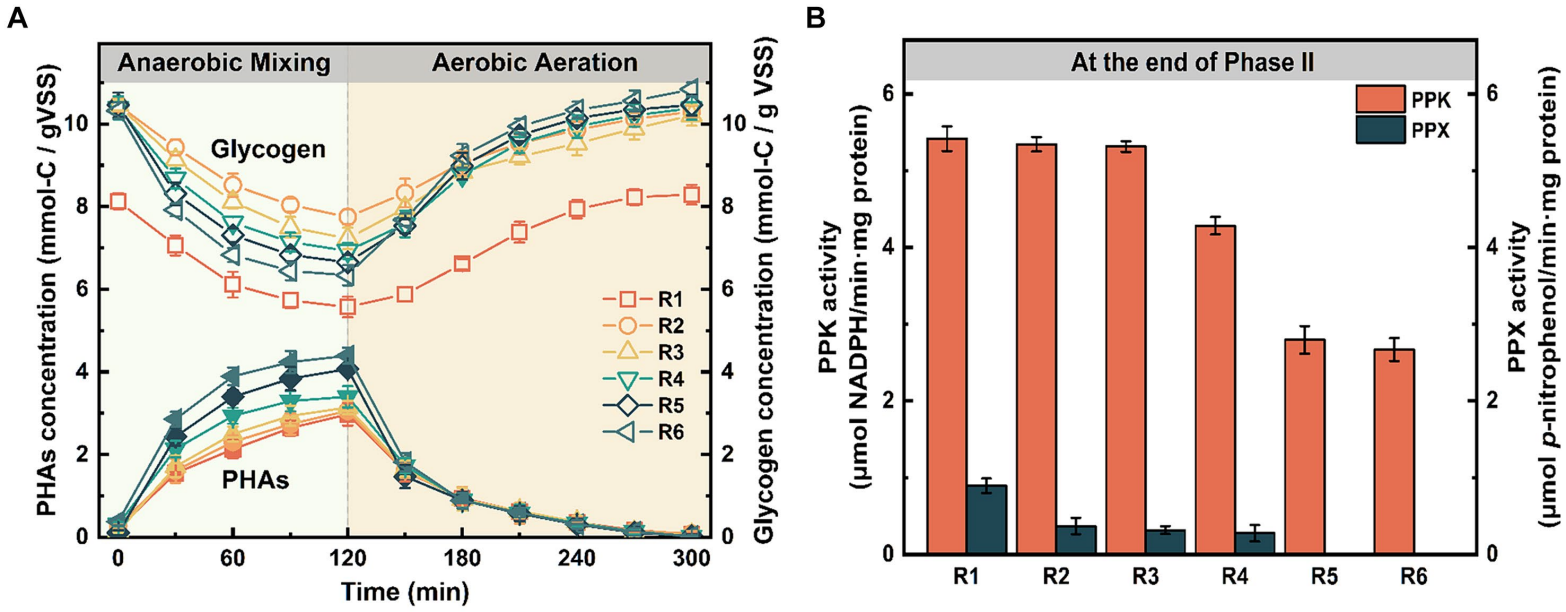
Effects of chronic Fe(III) exposure on **(A)** the intermediate products and **(B)** the activities of PPK and PPX. Error bars represent the standard deviation from three independent replicates.

To further evaluate changes in biological functions, the intermediate products of phosphorus metabolism were measured during a typical cycle under long-term Fe(III) stress (i.e., at the end of Phase II). From the periodic curves (Figure 4A), it was observed that the glycogen degradation rate induced by Fe(III) was significantly higher than in the control within the first 60 min after anaerobic mixing (*p* < 0.05). Moreover, we compared the ratios of PHA_synthesis_/VFA_uptake_, Gly_degradation_/VFA_uptake_, P_release_/VFA_uptake_, and Gly_synthesis_/VFA_degradation_ at the end of Phase II. Table 1 illustrates that the ratio of PHA_synthesis_/VFA_uptake_ increased simultaneously with the increasing mixture Fe content at the end of anaerobic mixing. Particularly, it’s noteworthy that the maximum ratio obtained in R6 reached 0.317 mol-C/mol-C. However, the ratio of P_release_/VFA_uptake_, as derived from Figures 1C,D, gradually decreased gradually from 0.285 mol-P/mol-C (control) to 0.024 mol-P/mol-C (R6). Correspondingly, a significant loss of aerobic phosphorus uptake was observed, despite the ratios of Gly_degradation_/VFA_uptake_ and Gly_synthesis_/PHA_degradation_ increased with rising Fe content (Table 1).

**Table 1:** Distribution of intermediate-related ratios at the end of Phase II.

Different systems	PHA_synthesis_/VFA_uptake_	Gly_degradation_/VFA_uptake_	P_release_/VFA_uptake_	Gly_synthesis_/VFA_degradation_
	(mol-C/mol-C)	(mol-C/mol-C)	(mol-P/mol-C)	(mol-C/mol-C)
R1	0.139	0.414	0.285	0.328
R2	0.116	0.436	0.232	0.385
R3	0.135	0.473	0.247	0.412
R4	0.157	0.598	0.152	0.469
R5	0.244	0.631	0.087	0.592
R6	0.317	0.687	0.024	0.614

Testing was performed in triplicate, and average means were used to apply the analysis.

The impact of prolonged Fe(III) stress on PPK and PPX activities was assessed (Figure 4B). After 155 days, PPK activity declined from 5.42 ± 0.16 μmol NADPH/(min·mg protein) in the control to low levels in R5 and R6 (2.79 ± 0.18 and 2.67 ± 0.15 μmol NADPH/(min·mg protein)). Moreover, PPX activity in the Fe(III)-treated groups notably lagged behind that in the control group (0.89 ± 0.09 p-nitrophenol/(min·mg protein)), and even no detectable PPX activity observed in R5 and R6 (Figure 4B).

### 3.4 Bacterial communities in Fe(III)-treated AS

**Figure 5:**
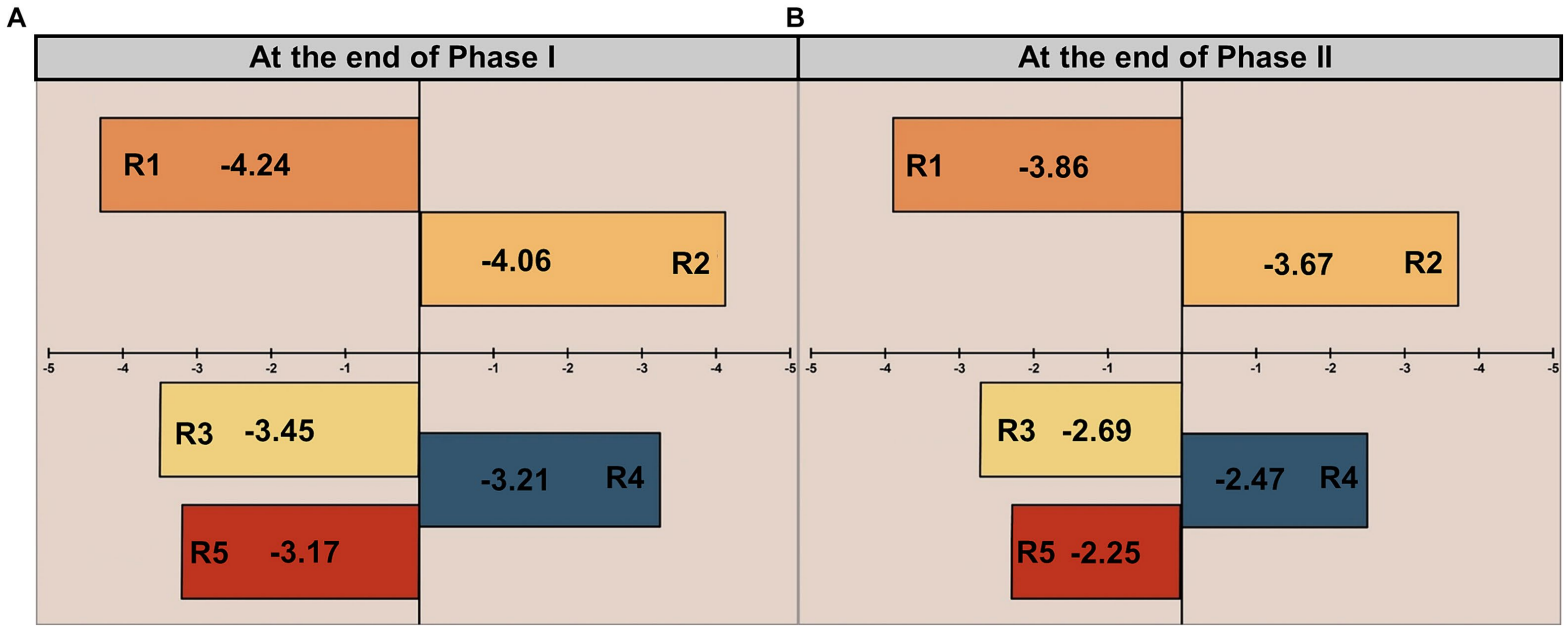
The relative contributions of deterministic process to community assembly at the end of **(A)** Phase I and **(B)** Phase II.

The functional bacterial community in AS with various influent Fe/P molar ratios was analyzed using Illumina MiSeq sequencing. Changes in microbial community diversity across different phases were assessed using the Shannon and Chao indices, as detailed in Supplementary Table S3. The coverage index exceeded 99% in all AS samples, indicating that the sequences adequately represented the majority of bacteria in each sample, and the sequencing depth was satisfactory for capturing most taxonomic diversity. To assess the similarity in Fe(III)-mediated bacterial communities, a community dynamics analysis was carried out using the Null model based on habitat associations developed by Stegen et al. (2012). Figure 5A presents the Beta Nearest Taxon Index (β-NTI) at the end of Phase I, which is −4.24 and − 4.06 in the low-Fe(III) groups (R2 and R3), and − 3.45, −3.21, −3.17 in the high-Fe(III) groups (R4, R5, and R6), respectively. The high-Fe(III) groups exhibited smaller phylogenetic distances relative to the low-Fe(III) groups. Despite the absolute value of β-NTI increasing at the end of Phase II, it remained below 2 in all AS samples (Figure 5B).

**Figure 6:**
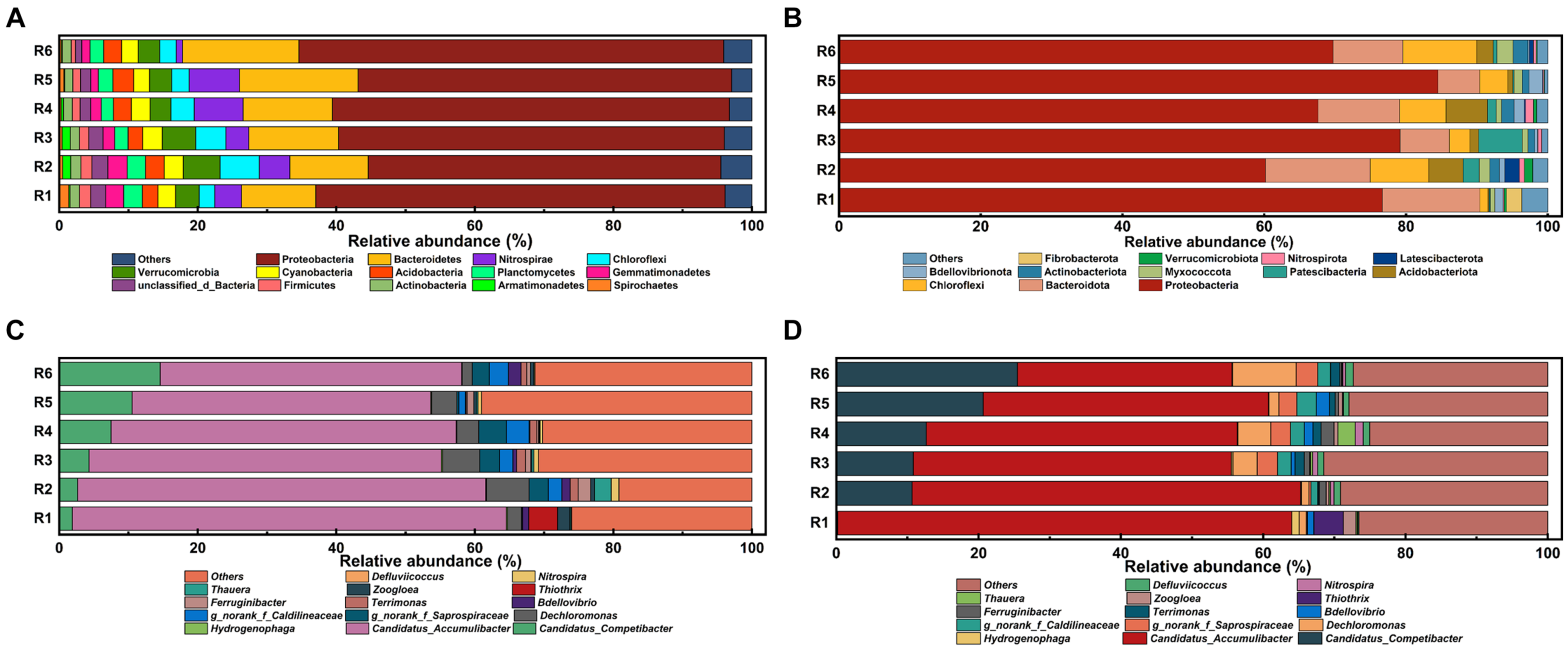
Overall bacterial community structures at phylum level at the end of **(A)** Phase I and **(B)** Phase II; abundances of the bacterial microbial communities at the genus level at the end of **(C)** Phase I and **(D)** Phase II. The minor bacteria with relative abundance lower than 1% were defined as “others”.

To elucidate the impact of prolonged Fe(III) stress on the phylogenetic diversity of AS samples, the taxonomic compositions of microbial communities were compared at both phylum and genus levels. The taxonomic analysis (Figure 6A) revealed that Proteobacteria dominated the microbial taxa at the phylum level at the end of Phase I, representing 50.89 to 61.32% (averaging 56.37%) of the microbiota. Bacteroidetes was also relatively abundant, with its relative abundance ranging from 10.73 to 17.10%. Additionally, despite their lower relative abundance (0.89 to 7.26%), Chloroflexi emerged as one of the dominant phylum among the common OTUs. However, following long-term operation, a significant (*p* < 0.05) and uneven shift in community composition between Fe(III)-treated and control groups was observed (Figure 6B). The average relative abundance of Proteobacteria increased from 56.37 to 72.95% by day 155. A reduction in the relative abundance of Bacteroidetes was noted in Fe(III)-treated groups except R1 and R2, with decrease rate ranging from 10.74 to 65.66%. Notably, the relative abundance of Chloroflexi, known to participate in glycolysis and sludge bulking processes, varied across the bioreactors, ranging from 1.15 to 10.40%.

The top 15 most abundant genus in each AS sample were identified (Figures 6C,D). At the genus level, *Candidatus Accumulibacter* was the most abundant PAO genus at the end of Phase I, representing 62.68, 58.93, 50.87, 49.81, 43.10, and 43.53% of the microbiota in the six SBRs, respectively. By the end of Phase II, the relative abundance of *Candidatus Accumulibacter* decreased from 62.89 to 60.83% in the control group, and notably from 43.53 to 30.15% in R6, aligning with the declining trend in phosphate removal efficiency (Figure 1D). Consistently, the relative abundance of *Candidatus Competibacter*, a common GAO, increased with rising Fe content in the mixture. At the same time, *Defluviicoccus* emerged as a predominant genus in the Fe(III)-treated groups, with a relative abundance of 1.10% in R6.

### 3.5 Key metabolic potentials of communities

**Figure 7:**
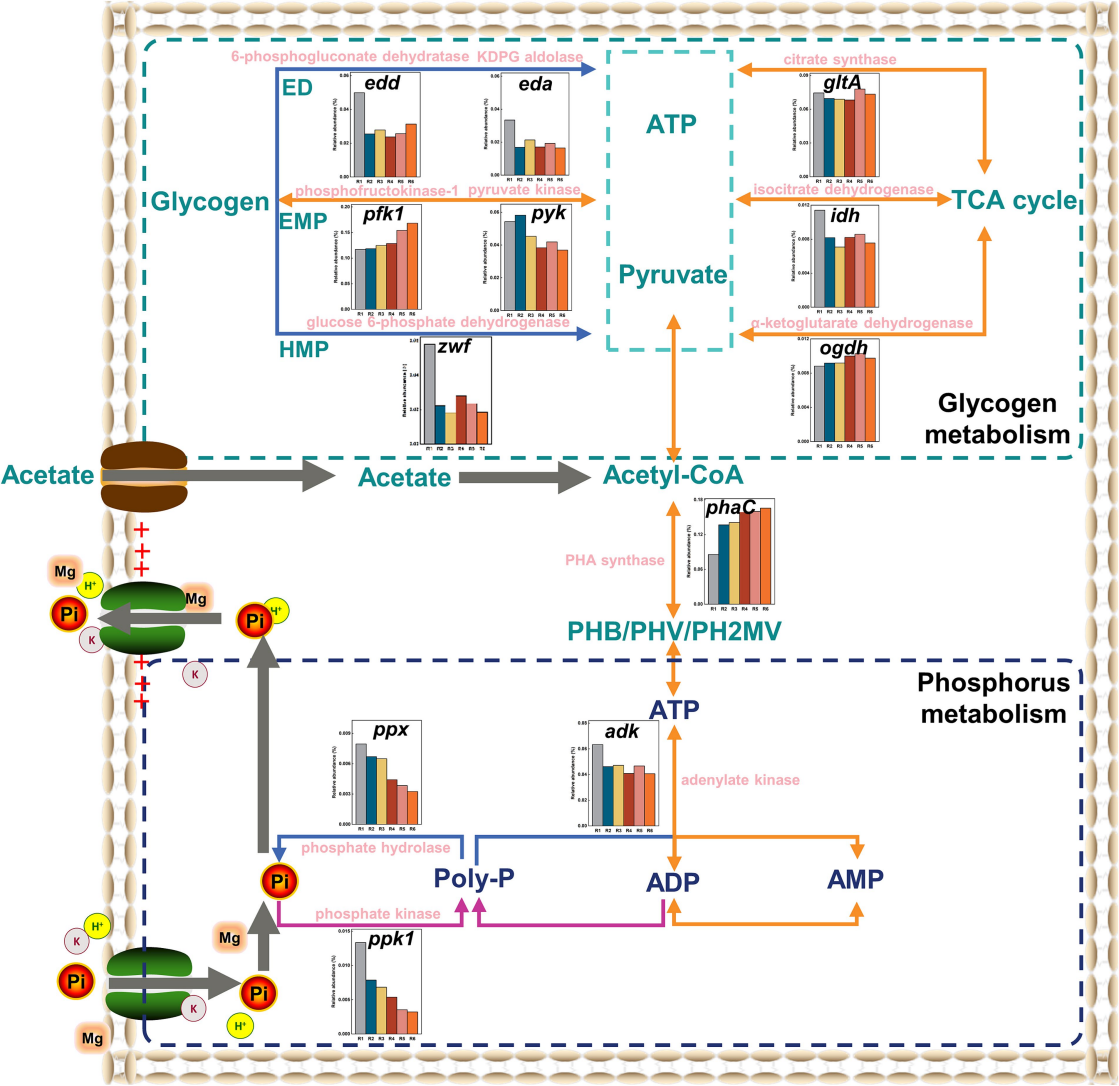
The variations in the abundance of key enzyme-encoding genes related to glycolysis and Poly-P accumulating in the SCPR system. The blue line denotes the metabolic process of anaerobic stage, the purple line denotes the metabolic process of aerobic stage, and the orange line denotes that the anaerobic and aerobic reactions were merged.

To explore the impacts of Fe(III) on key metabolic pathways for glycogen and phosphorus, metagenomic analyses were conducted. The metabolic potential for the whole community was profiled into individual functions that mediated specific pathways. Functional annotation analysis of glycogen and Poly-P metabolism-dependent genes was performed based on KEGG functional gene libraries. Generally, the glycogen metabolic pathways in bacterial cells mainly include the Embden–Meyerhof–Parnas (EMP), Entner–Doudoroff (ED), Hexose–Monophosphate (HMP) pathways, and the TCA cycle. The relative abundance of *pfk1*, a functional gene encoding phosphofructokinase-1, significantly increased with increasing Fe content (*p* < 0.05). Meanwhile, although the functional gene *pyk* (associated with pyruvate kinase) decreased relative to the control, the difference was not significant (*p* > 0.05) (Figure 7). Surprisingly, the relative abundance of *zwf*, the gene encoding 6-phosphate dehydrogenase, significantly decreased in the Fe(III)-treated groups compared to the control (*p* < 0.05). Additionally, the relative abundance of functional genes *gltA*, *ogdh*, and *idh* associated with citrate synthase, α-ketoglutarate dehydrogenase, and isocitrate dehydrogenase did not show substantive differences in the Fe(III)-treated groups (*p* > 0.05). Finally, the essential rate-limiting enzymes of the ED pathway, KDPG aldolase and 6-phosphogluconate dehydrogenase, a higher relative abundance of the encoded genes *eda* and *edd* in the Fe(III)-treated groups was observed compared to the control, consistent with the glycogen degradation rate in the anaerobic stage (Figure 4A).

In the phosphorus accumulating metabolism pathway, Figure 7 illustrates that the relative abundance of functional genes *ppk1* and *ppx* related to PPK and PPX decreased with increasing mixture Fe content. Gene abundance-based analysis did not detect changes in functional gene *pap* (associated with Poly-P AMP phosphotransferase). Furthermore, the relative abundance of *adk*, encoding adenylate kinase, was significantly higher in the control group than in the Fe(III)-treated groups (*p* < 0.05). This observation aligns with the highest PPK activity in the control group (Figure 4B). In contrast, the relative abundance of *phaC*, responsible for synthesizing PHAs from acetyl-CoA, was significantly higher in the Fe(III)-treated groups compared to the control (*p* < 0.05).

## 4 Discussion

### 4.1 Dynamics of the dominant phosphorus removal mechanism

Previous research has highlighted the cytotoxic effects of prolonged Fe(III) exposure on PAO cells, consequently inhibiting their phosphate release potential in AS (Smith et al., 2008; Liu et al., 2011). However, as shown in Figure 1A, Fe ions gradually accumulated in the supernatant when the sludge Fe content increased and reached a threshold level (Zhang et al., 2020). The Fe was primarily accumulated in the sludge phase during continuous operation. Given the weakly alkaline pH of the mixed solution, continuous Fe(III) dosing generated colloidal Fe(III) hydroxide with low solubility (Oikonomidis et al., 2010). These colloidal hydroxides provided numerous adsorption sites, exhibiting high adsorption and ion exchange properties towards phosphates (Wilfert et al., 2015). This observation was further supported by the distribution of phosphorus species in AS at the end of Phase II (Supplementary Figure S3A), revealing that phosphate bound to iron oxides constituted the major component of phosphorus species in AS from the experimental groups (78.06 ± 0.34% in R6). Consequently, influent phosphorus could also be captured by Fe(III) hydroxide via adsorption or complexation, hindering the aerobic phosphorus uptake process and thus inhibiting biological phosphorus removal.

In addition, Figure 1 shows that sludge phosphorus release was primarily affected by Fe content accumulation. The stimulation of phosphorus release due to Fe(III) addition could be a dose-dependent response, as observed under low-dose conditions (Figure 1C). However, the sludge phosphorus release was inhibited over the duration of exposure compared with the control (Figure 1D). The dose-response relationships between sludge Fe content and phosphorus release revealed low-dose stimulation and high-dose inhibition phenomenon. Therefore, the biological phosphorus removal was impeded with continuous Fe(III) dosing compared to the control due to low phosphate availability in the presence of sludge Fe accumulation (de Haas, 2000). In fact, phosphorus removal in the SCPR system is typically impacted by the co-regulation of biological metabolism and chemical adsorption. As a result, the phosphate removal capacity of high-dose groups (i.e., R4, R5, and R6) gradually recovered in the later Phase II, which was largely attributed to the fact that a large amount of Fe ion was not reduced (Crittenden et al., 2012). This also resulted in most of the phosphate in fresh sludge being chemisorbed by amorphous ferrihydrite (am-FeOOH) (Supplementary Figure S3B), and the adsorption procedure was allowed to equilibrate in a relatively short period of time (Zhang B. et al., 2019). These findings indicate that chemisorption became the primary phosphorus removal mechanism by competing with PAOs to bind phosphate after long-term Fe(III) exposure.

Despite a shift in the overall phosphorus removal mechanism, Fe-rich sludge exhibited limited phosphorus release capacity at the end of Phase II (Figure 1D). This phenomenon could be attributed to the recapture of phosphate released from intracellular or EPS by Fe ions, thereby forming poorly soluble compounds (Hu et al., 2019). However, other studies have suggested the existence of additional side effects that influence phosphorus release and uptake, and demonstrated that Fe(III) can reduce the total microbial biomass in AS (Ji et al., 2020). Hence, further exploration of the effects of Fe(III) on intracellular and extracellular bio-phosphorus has emerged as a crucial pathway for understanding potential inhibitory mechanisms.

### 4.2 Limitations on biological phosphorus release and uptake

Previously, studies have shown that Fe ions can bind to zoogloea, which contains numerous negatively charged functional groups such as carboxyl, phosphoryl, phenolic, and hydroxyl groups (Zhang et al., 2013). Importantly, as an important component of zoogloea, phosphorus in EPS was also affected by Fe(III) dosing. However, Figure 2A indicates that acute Fe(III) exposure does not cause any disturbance to phosphorus fate in EPS. Conversely, the decline in phosphorus levels in EPS with increasing sludge Fe content under chronic Fe(III) exposure suggests a cumulative effect of Fe(III) dosing. This trend appeared to correlate with the EPS protective mechanism, which protects microbial cells from metal toxicity by binding Fe ions (Wu et al., 2014). Consequently, excess Fe(III) resulted in a large aggregation of Fe species within EPS (Figure 2B). Fe ions can form complexes with macromolecular substances (such as short-chain Poly-P, proteins, and polysaccharides in EPS), subsequently transforming into co-precipitated or adsorbed products through EPS hydrolysis (Sheng et al., 2010). Protein inactivation and polysaccharide hydrolysis within the system further support this conclusion (Supplementary Figure S4) (Tang et al., 2018). Thus, in the high-dose Fe(III) exposure medium, the extracellular bio-phosphorus transport pathway was hindered after the 155-day exposure.

Regarding phosphorus transformation in PAO cells, micro-Fe dosing could enhance phosphorus release and uptake by promoting the effectiveness of phosphorus metabolism (Figure 3A) (Guo et al., 2010). However, excessive Fe(III) impaired PAO function. A decrease in intracellular phosphorus stocks suggests that Fe ions entering the PAO intracellular space result in impaired phosphorus transport and metabolism by inhibiting key enzyme activities (Li et al., 2018). This finding is consistent with the observation that the levels of functional genes encoding PPK and PPX are upregulated (discussed in section 3.3), ultimately reducing intracellular phosphorus storage (Figure 3B) (Huang et al., 2015; Zhang et al., 2017). Therefore, when intracellular Fe ions reached stability saturation, they exerted substantial cytotoxicity on PAOs, inhibiting their normal phosphorus removal function.

### 4.3 Transitions in the metabolic pathways of PAOs

The phylogenetic diversity (Figures 5A,B) confirms that bacterial community development was primarily governed by deterministic processes (Graham et al., 2016). In other words, long-term Fe(III) stress induced a succession of microbial communities toward dominant bacterial species. This finding was further supported by the taxonomic compositions of microbial communities at the genus level. PAO growth was partially impaired by chronic Fe(III) stress over time (Figure 6D), ultimately resulting in microbial succession from PAO populations to other populations (e.g., GAOs) (Ji et al., 2020). However, several less abundant bacteria harboring the *ppk1* and *ppx* genes were identified in AS samples, despite a continually declining abundance of *Candidatus Accumulibacter*. Certain genus, such as *Dechloromonas*, *Flavobacterium*, *Thauera*, *Zoogloea* and *Hydrogenophaga*, share the same phosphorus removal metabolic mechanism as widely reported PAOs, thereby facilitating efficient phosphorus removal and enrichment (Yan et al., 2022). This implied that bacteria with a typical PAO phenotype remained extensively active within microbial communities (Welles et al., 2015). Simultaneously, intracellular phosphorus release and uptake were hindered by chronic Fe(III) exposure (Figure 3B). Therefore, this led us to ask whether the evolution of dominant metabolic pathways was the major contributor to the decreased bio-phosphorus removal performance.

Changes in intermediate products (Figure 4A) and supernatant phosphate concentrations (Figure 1B) suggest that long-chain Poly-P in PAOs cells could serve as a prior energy source for phosphorus release in the control, whereas chronic Fe(III) exposure interfered with this microbial metabolism in other groups (Zeng et al., 2019). During the aerobic stage, excess Fe(III) can accelerate the glycogen synthesis rate with increasing Fe content in the mixture, resulting in more NADH production (Sun et al., 2017; Li et al., 2018). It is widely recognized that changes in PHAs and glycogen primarily depend on substrate concentrations and the microbial types that utilize them (Mino et al., 1998). As the major sources of influent COD were the same concentration of sodium acetate and glucose in all bioreactors, the accumulation and degradation of glycogen had a greater dependence on the dominant populations. Therefore, we hypothesized that this competitive advantage provided to microbial populations of non-dominant PAOs could potentially influence microbial succession patterns.

The uptake of external carbon sources and PHAs synthesis in PAOs during the anaerobic stage require sustained energy primarily derived from Poly-P hydrolysis (Oehmen et al., 2007). Conversely, GAOs, as competitors of PAOs, utilize anaerobic glycolysis to generate energy for carbon source uptake (Welles et al., 2015). Our findings revealed that Fe(III)-treated samples exhibited accelerated glycogen degradation rates and reduced key enzyme activities (diminished Poly-P synthesis and hydrolysis activities). This suggests a preference for glycolysis as the primary energy source in microbial communities, which aligns with the upregulation trend in the relative abundance of Chloroflexi (Figures 6A,B). Meanwhile, the energy generated from PHA oxidation was primarily utilized to expedite glycogen metabolism rather than to enhance phosphorus uptake (Table 1) (Mulkerrins et al., 2004; Acevedo et al., 2012). Based on these observations, it can be inferred that the metabolic pathway of PAOs likely transitions towards glycolysis-based metabolism from the initially dominant Poly-P hydrolysis under prolonged Fe(III) stress.

Figure 7 illustrates the upregulation of genes encoding key enzymes in the EMP pathway and the downregulation of genes encoding key enzymes in the HMP pathway under prolonged Fe(III) stress. This suggests that the EMP pathway could provide the reducing power and ATP for PHAs synthesis, aligning with the typical glycogen-accumulating metabolism (GAM) feature (Ni et al., 2022). The relative abundance of relevant functional genes in the TCA cycle did not show a significantly difference (*p* > 0.05), suggesting that the TCA cycle was rapidly activated in all samples to drive PHA synthesis. These results further support that the reducing power supplied by the TCA cycle in the anaerobic stage was not a decisive factor for glycogen metabolism (Acevedo et al., 2012). The upregulation of the relative abundance of the encoded genes *eda* and *edd* suggests that equal amounts of glycogen consumption allowed for faster ATP production through the ED pathway, compensating for the lack of reducing power and ATP supply in glycolysis. As a result, chronic Fe(III) exposure promoted a shift in the glycogen metabolic pathway and consequently PHAs synthesis. These collective findings indicate that the GAM pathway becomes more strongly expressed than the polyphosphate-accumulating metabolism (PAM) pathway with cumulatively increasing Fe levels, which brings about variations in the community phenotype.

Changes in functional genes *ppk1* and *ppx* indicate that Fe(III) suppressed biological phosphorus enrichment and removal through reducing functional gene levels encoding key rate-limiting enzymes (Ni et al., 2022). Observations of functional genes *adk* and *phaC* indicated that PAOs in the control obtained a competitive advantage by enhancing VFAs uptake, while PAOs in the Fe(III)-treated groups acquired a competitive advantage through glycolytic substitution (Acevedo et al., 2017; Zeng et al., 2019). This analysis affirmed again that chronic Fe(III) exposure inhibited PAM and enhanced GAM. Consequently, PAOs transferred their metabolic pathways from PAM to GAM as a survival strategy under chronic Fe(III) stress, which was the underlying cause of the decreased biological phosphorus removal efficiency.

## 5 Conclusion

Based on the above results and discussions, a potential inhibitory pathway of Fe(III) on biological phosphorus removal in AS is proposed. Initially, a significant portion of phosphorus in the system had chemically precipitated (or adsorbed) with accumulated Fe in the sludge mixture, leading to a decreased availability of phosphorus for bio-metabolism. Subsequently, PAOs remained a predominant genus in the SCPR system, despite a decline in their abundance with increasing sludge Fe content. However, the majority of PHAs consumed during PAOs metabolism primarily replenished glycogen rather than facilitating phosphorus uptake, resulting in the elimination of their phosphorus release function. Consequently, the metabolic pathway of communities shift from PAM to GAM played a decisive role in the reduction of biological phosphorus removal efficiency under chronic Fe(III) stress.

## Data availability statement

The data presented in the study are deposited in the NCBI Sequence Read Archive repository, accession number PRJNA1104697.

## Author contributions

YH: Conceptualization, Data curation, Methodology, Writing – original draft, Visualization. HC: Conceptualization, Data curation, Writing – review & editing, Methodology. XH: Conceptualization, Methodology, Project administration, Writing – review & editing. LL: Conceptualization, Writing – review & editing, Data curation, Formal analysis, Visualization. QH: Conceptualization, Supervision, Writing – review & editing, Funding acquisition, Resources.
