# Supplementary material for: Inhibition of phosphorus removal performance in activated sludge by Fe(III) exposure: transitions in dominant metabolic pathways
Source: Front Microbiol. 2024 Jun 12;15:1424938. doi: 10.3389/fmicb.2024.1424938 (PMC11201142; doi:10.3389/fmicb.2024.1424938)
Supplement: Supplementary file 1 [file Data_Sheet_1.docx]

Supporting Information

For

**Inhibition of Phosphorus Removal Performance in Activated Sludge by Fe(III) Exposure: Transitions in Dominant Metabolic Pathways**

Yiyihui Hong, Hong Cheng, Xiaoliu Huangfu, Qiang He*, Lin Li*

Key Laboratory of the Three Gorges Reservoir Region’s Eco-environment, Ministry of Education, College of Environment and Ecology, Chongqing University 400044, China.

*^*^*Corresponding author

E-mail address: [heqiang@cqu.edu.cn](mailto:heqiang@cqu.edu.cn), [li.lin@cqu.edu.cn](mailto:li.lin@cqu.edu.cn)

The Number of Pages: 6

The Number of Text: 3

The Number of Figure: 4

The Number of Table: 3

**Text S1.** Reactor operating conditions.

A laboratory-scale sequencing batch reactor (SBR) was selected as the experimental setup to ensure result comparability. The SBR, constructed of clear Plexiglas, measures 12 cm in length with an inner diameter of 10 cm. It has a total volume of 3.5 L, with an effective volume of 2.5 L. Flow control of influent and effluent was managed using electromagnetic flow meters and valves. Ports for effluent, influent, and sludge discharge were positioned vertically on one side of the reactor. The outlet height was placed at the intermediate position, the inlet at the top, and the sludge discharge at the bottom.

**Text S2.** Reactor operating procedures.

During the feeding phase, 1.25 L of synthetic wastewater containing varying concentrations of Fe(III) was introduced into the SBRs via a peristaltic pump. Throughout the anaerobic mixing stage, a magnetic stirrer controlled the rotor, maintaining a stirring speed of 180-210 r/min. Aeration was conducted by supplying 1.2-1.5 L/min of air through a porous diffuser to uphold dissolved oxygen (DO) levels between 2 and 3 mg/L. Additionally, to ensure adequate solution mixing during the aerobic phase, a magnetic stirrer operated at a slower speed of 80-120 r/min. At the end of each aeration cycle, 65 mL of mixture was discharged, resulting in a solid retention time of approximately 10 days and an initial mixed liquor suspended solids (MLSS) range of 3800-4200 mg/L. Decanting occurred during the decanting phase, with half of the supernatant volume being decanted at the end of each cycle, resulting in an actual hydraulic residence time of 12 hours.

**Text S3.** Sequence quality control, genome assembly, genes prediction and abundance calculation procedures.

Quality filtering was performed using Fastp (Version 0.20.0) to eliminate low-quality reads with a quality value lower than 20, length shorter than 50 bp, or containing ambiguous bases. Following quality filtering, high-quality paired-end reads from all samples were assembled using Megahit (Version 1.1.2), with a minimum contig length of 350 bp. MetaGene was employed for open reading frame (ORF) prediction based on assembled contigs, selecting genes with a nucleic acid length longer than 100 bp for translation into amino acid sequences. To cluster predicted gene sequences, a non-redundant gene set was constructed using CD-HIT with >95% sequence identity and >0.9 coverage across all sequences. Subsequently, high-quality reads (>95% identity) from each sample were aligned to representative genomes using SOAPaligner (Version 2.21), and gene abundance was calculated using the Reads Per Kilobase Million (RPKM) method.


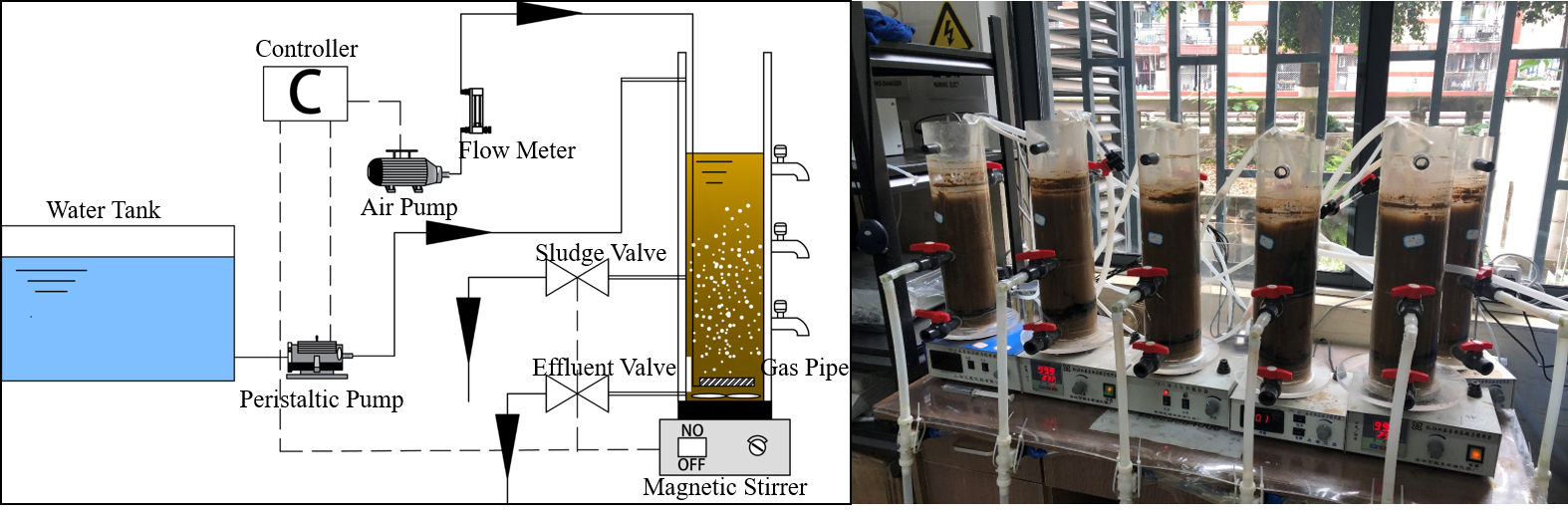


**Figure S1.** The construction and flow chart of the applied SBR.


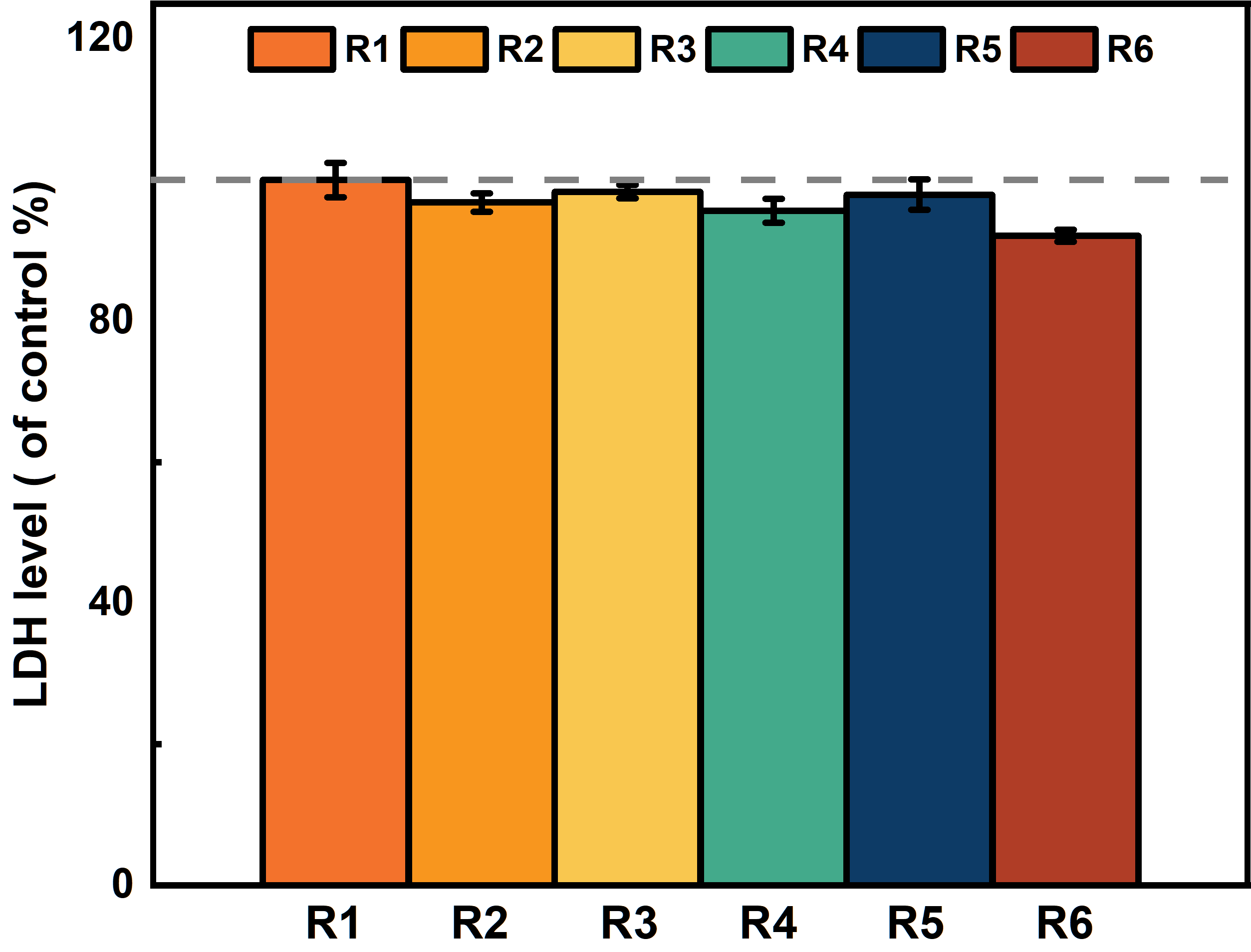


**Figure S2.** Effects of Fe(III) on LDH release ROS production at the end of Phase II. Error bars represent the standard deviation from three independent replicates.


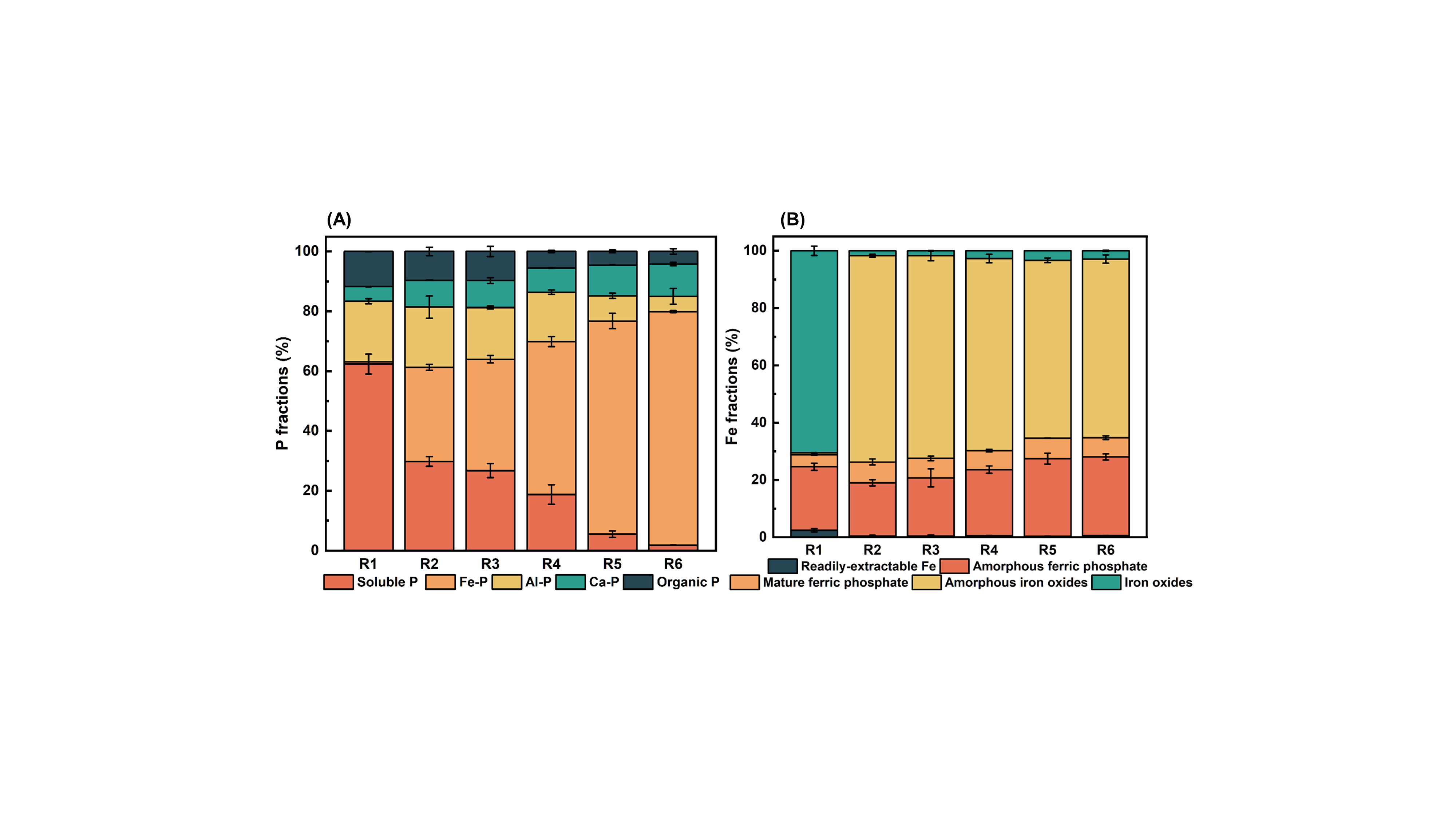


**Figure S3.** (A) P and (B) Fe fractions extracted from sludge samples. Error bars represent the standard deviation from three independent replicates.


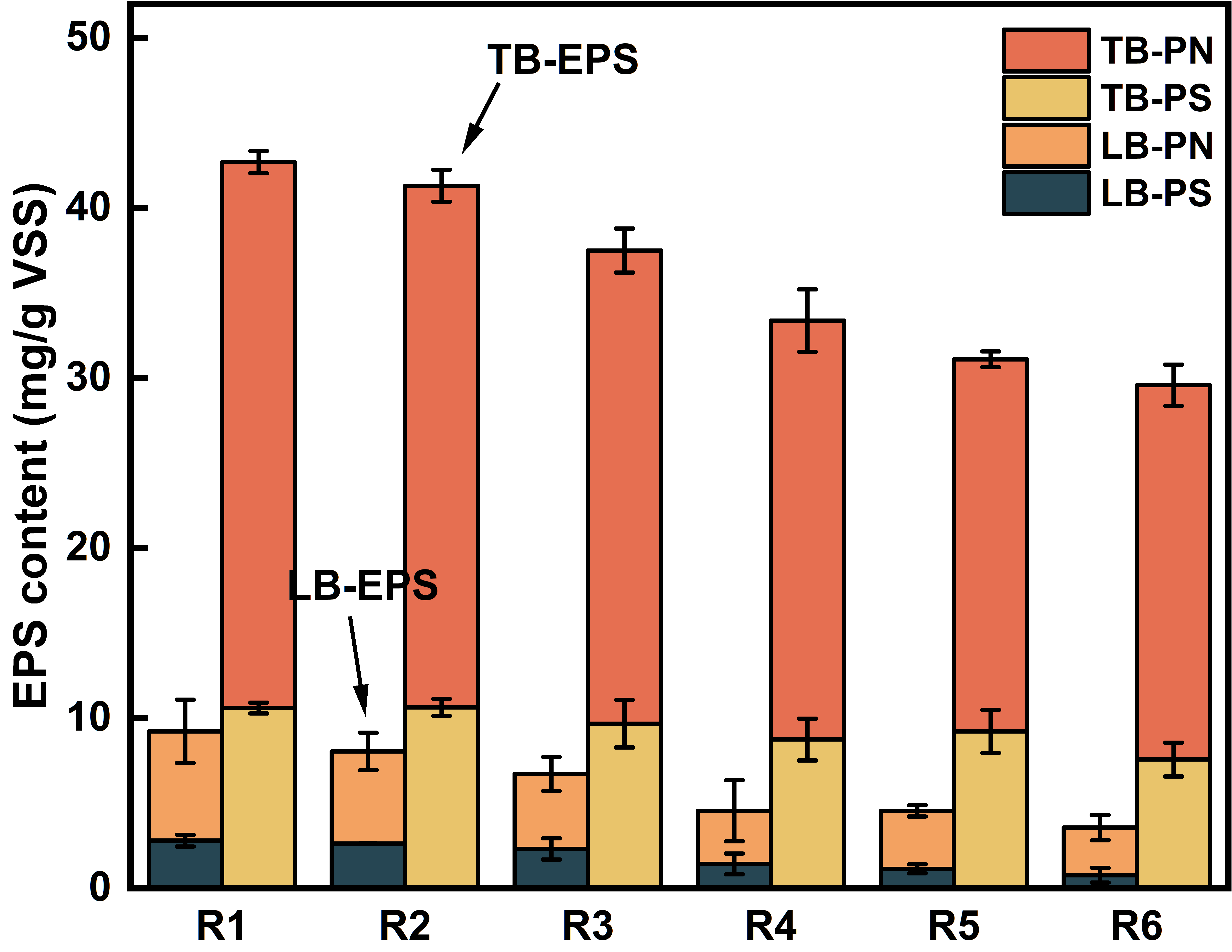


**Figure S4.** Distribution of EPS component at the end of Phase II. Error bars represent the standard deviation from three independent replicates.

**Table S1**. Water quality characteristics of the synthetic wastewater.

| Composition | Content | Characteristics |
| --- | --- | --- |
| CH_3_COONa | 350 mg/L | COD 400 mg/L |
| C6H12O6 | 150 mg/L |  |
| NH_4_Cl | 76.4 mg/L | Ammonia Nitrogen 20 mg/L |
| KH_2_PO_4_ | 21.5 mg/L | Total Phosphorus 5 mg/L |
| CaCl_2_ | 5 mg/L |  |
| MgSO_4_·7H_2_O | 10 mg/L |  |
| Stock Solution | 0.5 mL/L |  |
| HCl or NaOH | 0.5 mol/L |  |

**Table S2**. Ingredients of the trace element stock solution.

| Composition | Content | Composition | Content |
| --- | --- | --- | --- |
| H_3_BO_3_ | 150 mg/L | Na_2_MoO_4_·2H_2_O | 60 mg/L |
| CuSO_4_·5H_2_O | 30 mg/L | ZnSO_4_·7H_2_O | 120 mg/L |
| KI | 180 mg/L | CoCl_2_·6H_2_O | 150 mg/L |
| MnSO_4_·H_2_O | 120 mg/L | EDTA-2Na | 10000 mg/L |

**Table S3**. Diversity indices of microbial communities at different experimental phases.

|  | Sample | Shannon | Chao | Coverage |
| --- | --- | --- | --- | --- |
| Phase I | R1 | 4.11 | 457.9 | 99.31% |
|  | R2 | 6.59 | 675.15 | 99.75% |
|  | R3 | 3.91 | 488.26 | 99.38% |
|  | R4 | 3.22 | 635.77 | 99.54% |
|  | R5 | 2.98 | 493.67 | 99.77% |
|  | R6 | 3.14 | 621.87 | 99.46% |
| Phase II | R1 | 2.26 | 467.86 | 99.60% |
|  | R2 | 4.2 | 652.16 | 99.29% |
|  | R3 | 3.15 | 496.79 | 99.37% |
|  | R4 | 3.92 | 605.22 | 99.83% |
|  | R5 | 2.13 | 453.21 | 99.75% |
|  | R6 | 3.47 | 598.93 | 99.80% |
| Phase III | R1 | 2.09 | 382.63 | 99.46% |
|  | R2 | 3.53 | 395.48 | 99.23% |
|  | R3 | 2.94 | 438.15 | 99.81% |
|  | R4 | 3.01 | 368.53 | 99.79% |
|  | R5 | 2.08 | 381.33 | 99.23% |
|  | R6 | 2.24 | 379.26 | 99.60% |
